# Expression of recombinant *Mycobacterium bovis *antigen 85B by *Mycobacterium smegmatis *mc^2^

**DOI:** 10.1186/1753-6561-8-S4-P169

**Published:** 2014-10-01

**Authors:** Amanda Soares, Caroline Rizzi, Ana carolina Peiter, Julia Labonde, Odir Antonio Dellagostin

**Affiliations:** 1 Universidade Federal de Pelotas, Centro de Desenvolvimento Tecnológico, Pelotas, RS, Brazil

## Background

Bovine tuberculosis (TB) is a disease which constitutes an important economic and zoonotic problem in Latin America. The etiological agent, *Mycobacterium bovis*, has several antigens described and well characterized. The *M. bovis *antigen 85B (Ag85B), encoded by *fbpB *gene, is the bacillus immunodominant antigen and promising target for vaccine and diagnosis development [1]. Ag85B combined with other immunogens is considered as a potential diagnostic tool for TB ELISA assay [2]. Anti-Ag85B antibodies have high sensitivity and specificity for mycobacteria detection in commercial tests [3]. Here we describe the recombinant Ag85B production by *Mycobacterium smegmatis*, a fast growing mycobacterial specie.

## Methods

Two pairs of synthetic oligonucleotide primers were designed using the Vector NTI software, based on the *M. bovis *AF2122/97 complete genome sequence. The histidine tail codons were included in the reverse primers in order to facilitate recombinant proteins purification by nickel affinity columns. These primers were used for PCR amplication of *fbpB *gene, including its own promoter (1500 bp) and one immunodominant portion (873 bp) of the same gene. The PCR products were digested with *Kpn*I, and *Xba*I and *Hin*dIII, respectively. The digestion products were then cloned into the respective mycobacterial expression vectors: pUP410 and pUS2000, which were previously digested with the same restriction enzymes. The ligations were transformed by electroporation into *E. coli *TOP 10 competent cells and transformed cells were selected on LB agar medium containing kanamycin. The recombinant plasmids were extracted using the QIAprep ^® ^Spin Miniprep Kit (Qiagen). To confirm the *fbpB *gene cloning, the recombinant vectors were digested with *Xho*I restriction enzyme, which has a restriction site within the cloned fragment and vectors. *M. smegmatis *mc^2 ^eletrocompetent cells were transformed with recombinant vectors, grown for 3 hours at selective medium (7H9 medium supplemented with 10% OADC, 0.2% glycerol and 0.05% Tween 80) containing kanamycin. The cultures were centrifuged, the pellet suspended in 50 mM Tris-HCl buffer and lysed by sonification. The soluble and insoluble fractions were separated by centrifugation. The Ag85B expression was detected by western blot employing mouse polyclonal anti-Ag85B and anti-histidine antibodies.

## Results and conclusions

The internal cleavage of recombinants vectors by *Xho*I confirmed the *fbpB *cloning into pUP410 and pUS2000. The recombinant antigen expression with expected molecular mass was observed in the soluble fraction of recombinant *M. smegmatis*. When the expression was compared to negative controls (cell extracts of BCG Pasteur and *M. smegmatis *mc^2^), we observed a more pronounced Ag85B expression at western blot employing mouse polyclonal anti-Ag85B. High levels of expression of Ag85B occurred because pUP410 and pUS2000 have strong mycobacterial promoters and are epissomal vectors which are present in number of five copies per transformed cell [4]. Moreover, western blot employing anti-histidine antibodies demonstrated antigen expression at recombinant *M. smegmatis*, but not at negative controls. The recombinant antigen production by *M. smegmatis *will enable the subsequent purification and production of polyclonal and monoclonal antibodies against Ag85B.
